# Five years follow up of buprenorphine treatment in Clinical Center of Montenegro

**DOI:** 10.1192/j.eurpsy.2023.1376

**Published:** 2023-07-19

**Authors:** I. Ljutica, Z. Barac Otasevic

**Affiliations:** 1 Addiction branch, Psychiatric Clinic of Clinical Center of Montenegro; 2 addiction branch, Poliklinika Filipovic, Podgorica, Montenegro

## Abstract

**Introduction:**

Montenegro is a country in Southeastern Europe. Podgorica, the capital and largest city covers 30 % of its total population of 621 000 and around 80 % of patients enrolled in buprenorphine treatment.

Buprenorphine was registered in Montenegro in May 2017.

Buprenorphine is an opioid partial agonist. It’s a safe and an effective option for the treatment of opioid addiction. Buprenorphine may be abusable. Its abuse potential, however, is lower in comparison with that of opioid full agonists.

**Objectives:**

Benefits of buprenorphine treatment:

Suppress symptoms of opioid withdrawal. Reduce illicit opioid use. Help patients stay in treatment.

Buprenorphine maintenance keeps the person stable while they make positive changes in their lives.

Health problems are reduced or avoided, especially those related to injecting, such as HIV, hepatitis B and hepatitis C viruses, skin infections and vein problems.

Crime behavior reduction

**Methods:**

Clinical study.

**Results:**

We followed the patients who were enrolled in the buprenorphine substitution treatment for a period of five years, to be more precise in the period from May 2017 August 2022.

In May of 2017 only 33 patients were enrolled in program, the number of patients were gradually increased to 193 patients in December of 2017. Until the December of 2018 the number of patients was increased to 203. In December of 2019 numbers of patients were increased to 291. At the end of 2020 numbers of patients were 348. In December of 2021 numbers of patients were increased to384 and in August of 2022 numbers of patient were 426.

**Conclusions:**

Health problems are reduced or avoided.

Crime behavior was reduced for 65% over five year’s period.

A total of 124 new cases of HCV infection were discovered from the beginning of study to the end of July of 2022.

Doses are required only once a day.

For most adults with opioid use disorder, maintenance therapy with buprenorphine is the most effective treatment approach.

**Disclosure of Interest:**

None Declared

